# The dual PI3K/mTOR inhibitor GSK2126458 is effective for treating solid renal tumours in *Tsc2*^+/-^ mice through suppression of cell proliferation and induction of apoptosis

**DOI:** 10.18632/oncotarget.17215

**Published:** 2017-04-19

**Authors:** Kalin Narov, Jian Yang, Paulina Samsel, Ashley Jones, Julian R Sampson, Ming Hong Shen

**Affiliations:** ^1^ Institute of Medical Genetics, Division of Cancer and Genetics, School of Medicine, Cardiff University, Heath Park, Cardiff CF14 4XN, UK

**Keywords:** tuberous sclerosis, mTOR, rapamycin, GSK2126458, renal tumours

## Abstract

Tuberous sclerosis (TSC) is an inherited tumour syndrome caused by mutations in *TSC1* or *TSC2* that lead to aberrant activation of mTOR. Tumour responses in TSC patients to rapamycin, an allosteric inhibitor of mTOR, or its analogs are partial and reversible probably due to feedback activation of Akt. In this study, we examined the efficacy of GSK2126458, an ATP-competitive dual inhibitor of PI3K/mTOR, in comparison to rapamycin for treatment of renal tumours in genetically engineered *Tsc2*^+/-^ mice. We found that both GSK2126458 and rapamycin caused significant reduction in number and size of solid renal tumours. GSK2126458 also significantly reduced the number and size of all lesions (cystic, papillary and solid) although to a lesser extent compared to rapamycin. GSK2126458 inhibited both PI3K and mTOR while rapamycin exerted stronger inhibitory effect on mTORC1 in renal tumours. Furthermore, GSK2126458 and rapamycin suppressed proliferation of tumour cells. Importantly, GSK2126458 increased apoptosis of solid tumours but rapamycin did not. Further investigations are therefore needed to test whether rapamycin in combination with GSK2126458 could promote apoptosis and thus improve therapy of TSC-associated renal tumours.

## INTRODUCTION

Tuberous sclerosis (TSC) is an inherited disease caused by mutations in *TSC1* or *TSC2* that lead to aberrant activation of mTOR and development of tumours in multiple organs. TSC patients frequently develop renal manifestations, usually multiple and bilateral angiomyolipomas (AML) that are the leading cause of adult deaths from the disease. Renal cysts are also commonly observed and renal cell carcinoma (RCC) develops in around 2% of TSC patients [1]. Treatment with the mTOR inhibitor rapamycin (sirolimus) or its derivative everolimus significantly reduces the size of renal AML in TSC patients [2–4]. Everolimus has also demonstrated clinical efficacy in TSC-associated renal carcinoma [5]. However, responses of AML and other TSC-associated tumours to these mTOR inhibitors are partial and tumours that initially respond to treatment usually regrow after drug withdrawal. Partial resistance to rapamycin or its derivatives is suggested to be associated with loss of negative feedback regulation that leads to increased phosphorylation and activation of AKT [6].

To prevent feedback activation of Akt, ATP-competitive dual inhibitors of PI3K/mTOR have been developed and used in clinical trials for cancer therapy [7]. GSK2126458 is a highly potent and orally bioavailable dual ATP-competitive inhibitor of PI3K and mTOR [8]. Preclinical studies suggest that GSK2126458 inhibits proliferation of a variety of cancer cell lines and tumours in xenograft mouse models with induction of apoptosis and attenuation of the PI3K/Akt/mTOR activity [8, 9]. A recent phase I clinical trial indicates that GSK2126458 is well tolerated in patients across multiple solid malignancies. Tumour responses and disease stabilization were documented in patients with several tumour types including bladder and renal cell carcinoma [10].

*Tsc2*^+/-^ mice spontaneously develop various lesions in the kidneys including cysts, papillary adenomas, solid adenomas and carcinomas [11]. These lesions are associated with aberrant activation of the PI3K/Akt/mTOR signalling pathway [12]. In this study, we tested the efficacy of GSK2126458 compared to rapamycin for treatment of renal lesions in *Tsc2*^+/-^ mice.

## RESULTS

### Activation of oncogenic pathways in renal tumours of *Tsc2*^+/-^ mice

**Figure 1:**
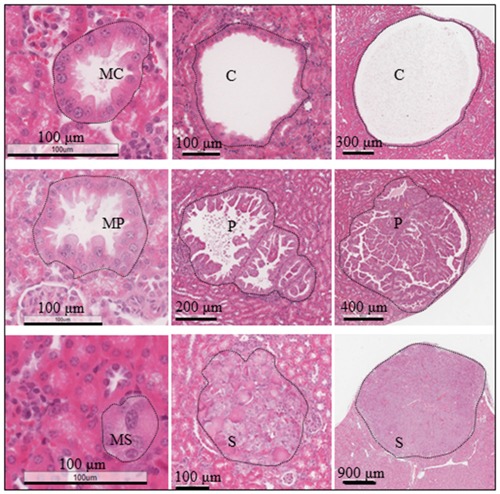
Renal tumours in *Tsc2*^+/-^ mice Kidney sections were prepared from 14 months old *Tsc2*^+/-^ mice and HE-stained. Representative renal lesions including micro-lesions (micro-cystic, MC; micro-papillary, MP; micro-solid, MS also referred to as microscopic intraepithelial neoplasia), cysts (C), papillary adenomas (P), and solid tumours (S). Black lines are scale bars.

**Figure 2:**
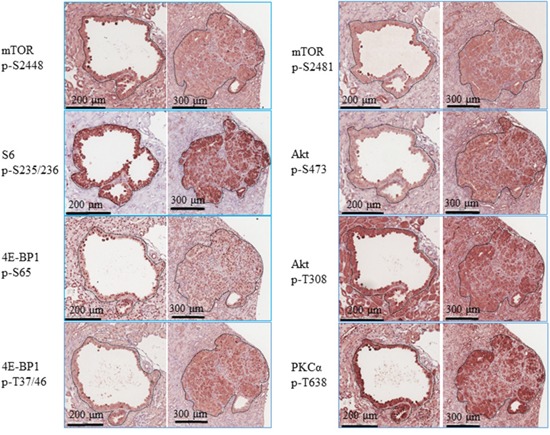
PI3K/Akt/mTOR signalling in renal tumours of *Tsc2*^+/-^ mice Kidney sections prepared from 14 months old *Tsc2*^+/-^ mice were used for IHC analysis. Representative IHC-stained sections were presented to show phosphorylation of mTOR at S2448 and S2481, S6 at S235/236, 4E-BP1 at T37/46 and S65, and Akt at T308 and S473 in renal tumours. Twenty cystic and 20 solid renal lesions from 10 *Tsc2*^+/-^ mice were analysed consistently showing similar protein phosphorylation levels. Black lines are scale bars.

The kidneys of adult *Tsc2*^+/-^ mice developed lesions including cysts, papillary adenoma, solid adenoma and carcinoma (Figure 1). Lesions varied in size from microscopic intraepithelial neoplasias to macroscopic carcinomas. Tumour cells were larger than adjacent normal cells and had hyper-chromatic enlarged nuclei. Lesions had aberrant activation of the mTOR complex 1 (mTORC1) and mTORC2 as evidenced by the increased phosphorylation of mTOR, Akt, PKCα, S6 and 4E-BP1 compared to the adjacent normal tissues (Figure 2). In addition to mTOR, Akt has many other substrates [13]. Akt substrates (RXXS*/T*) and Akt substrates (RXRXXS*/T*) were highly phosphorylated in renal lesions (Supplementary Figure 1). The MAPK pathway was also activated in these lesions as indicated by increased phosphorylation of RAF1 and Erk1/2 (Supplementary Figure 2).

### Efficacy of GSK2126458 and rapamycin on renal tumours of *Tsc2*^+/-^ mice

**Figure 3:**
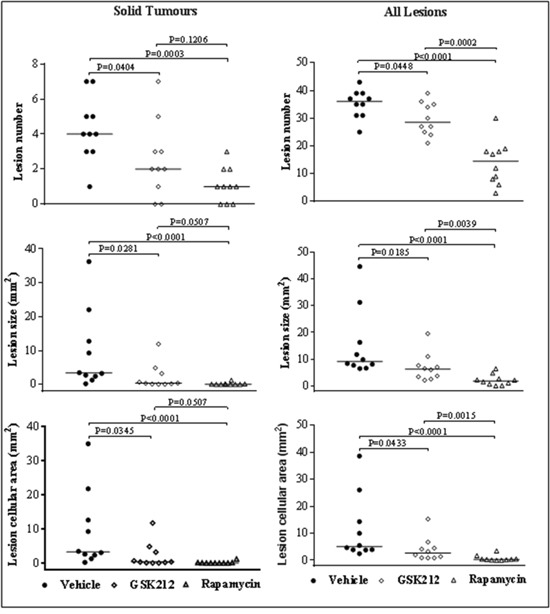
Efficacy of GSK2126458 and rapamycin on renal tumours in *Tsc2*^+/-^ mice *Tsc2*^+/-^ mice were treated from 12 months old for 2 months (n=10 each group). Mice were sacrificed for tumour burden assessment at the age of 14 months. Dosages are described in methods. Kidney sections were prepared for histological assessment of treatment efficacy. **Left panel**: comparison of total number and size (area) as well as cellular area of all lesions (cystic, papillary and solid). **Right panel**: comparison of total number and size (area) as well as cellular area of solid tumours. Horizontal bars indicate a median. For detailed statistical analysis see Supplementary Tables 1 and 2.

To test the anti-tumour efficacy of GSK2126458, we first determined the maximum tolerated dose (MTD) of GSK2126458 in *Tsc2*^+/-^ mice in a two weeks’ pilot study. Daily doses greater than 2 mg/kg of GSK2126458 via intraperitoneal injection caused significant loss of body weight or diarrhoea or both, with symptoms in female *Tsc2*^+/-^ mice more severe than in males. This maximum tolerated dose was therefore used in this study. For comparison, 3 groups of 10 mice each were treated for two months from the age of 12 months with vehicle, GSK2126458 (2 mg/kg) or rapamycin (4 mg/kg). Tumour burden was compared by analysing both solid renal tumours only and renal lesions of all types. Both GSK2126458 and rapamycin significantly reduced total number (P=0.0404; P=0.0003), size (P=0.0281; P<0.0001) and cellular area (P=0.0345; P<0.0001) of solid renal tumours (Figure 3, Supplementary Table 1). Similarly, both GSK2126458 and rapamycin significantly reduced total number (P=0.0448; P<0.0001), size (P=0.0185; P<0.0001) and cellular area (P=0.0433; P<0.0001) of all lesion types (Figure 3, Supplementary Table 2). Rapamycin caused greater reduction than GSK2126458 in number (P=0.0002), size (P=0.0039) and cellular area (P=0.0015) of all types of lesions but the difference in reduction of solid tumour burden was not significant between GSK2126458 and rapamycin treated mice (Figure 3, supplementary Tables 1 and 2).

### Effect of GSK2126458 and rapamycin on PI3K/Akt/mTOR and Erk1/2 signalling in renal tumours of *Tsc2*^+/-^ mice

**Figure 4:**
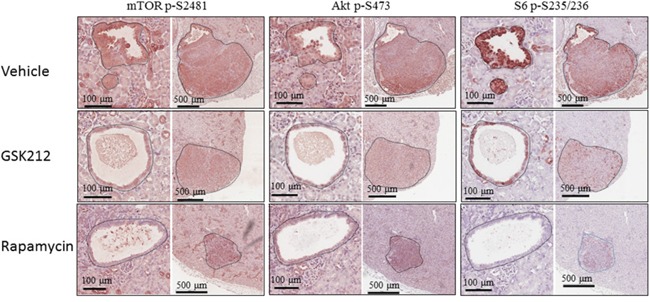
Effect of GSK2126458 and rapamycin on mTOR signalling of renal tumours in *Tsc2*^+/-^ mice Kidney sections prepared from 14 months old *Tsc2*^+/-^ mice treated with vehicle, GSK2126458 or rapamycin were used for IHC analysis. Representative IHC-stained sections were presented to show phosphorylation of mTOR at S2481, S6 at S235/236 and Akt at S473 in renal tumours. Black lines are scale bars.

**Figure 5:**
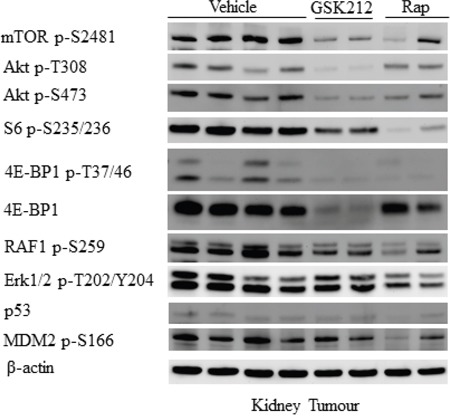
Effect of GSK2126458 and rapamycin on mTOR/MAPK/apoptotic signalling of renal tumours in *Tsc2*^+/-^ mice Western blot was used to analyse mTOR/MAKP/apoptotic signalling. Proteins were prepared from solid renal tumours dissected from *Tsc2*^+/-^ mice treated for one month with vehicle, GSK2126458 or rapamycin. Beta-actin was used as a loading control. Representative Western blots were presented to show phosphorylation of mTOR at S2481, Akt at T308 and S473, S6 at S235/236, 4E-BP1 at T37/46, RAF1 at S256, Erk1/2 at T202/Y204 and MDM2 at S166 as well as expression of 4E-BP1 and p53.

Kidney sections from the mice of different treatment groups were used to investigate PI3K/Akt/mTOR signalling with IHC. Phosphorylation of S6 at S235/236 is one of the key indicators for mTORC1-dependent signalling and phosphorylation of mTOR at S2481 and of Akt at S473 are two important indicators of mTORC2-dependent signalling. As shown in Figure 4, decreased phosphorylation of mTOR at S2481, Akt at S473 and S6 at S235/236 was observed in renal lesions of GSK2126458 treated mice. Phosphorylation of these proteins was also reduced in renal lesions of rapamycin treated mice (Figure 4). Rapamycin exhibited a greater inhibitory effect on S6 phosphorylation than GSK2126458. Further examination of additional IHC stained kidney sections revealed remarkable variations in Akt phosphorylation at S473 in rapamycin treated renal lesions, and 57% (20/35) of cysts treated by rapamycin had highly phosphorylated Akt at S473 while GSK2126458 consistently decreased Akt phosphorylation in all lesions (Supplementary Figure 3). However, no increased phosphorylation of Akt at S473 was observed in solid tumours of rapamycin treated mice compared to vehicle. Consistent with these observations, Western analysis demonstrated that after treatment with GSK2126458 solid renal tumours had decreased phosphorylation of these proteins at the specified serine sites, although GSK2126458 had greater inhibitory effect on Akt S473 and rapamycin on S6 S235/236 (Figure 5). GSK2126458 reduced expression of 4E-BP1 while both GSK2126458 and rapamycin attenuated phosphorylation of 4E-BP1 at T37/46 (mTORC1 indicator) in solid tumours (Figure 5). In addition, GSK2126458 reduced phosphorylation of Akt at T308 but rapamycin did not in solid tumours (Figure 5). These results suggest that GSK2126458 inhibited both PI3K and mTOR in all types of renal lesions and that its effects were greater on mTORC2 but less on mTORC1 than rapamycin in solid tumours. Furthermore, GSK2126458 appeared to reduce phosphorylation of RAF1 at S259 but not Erk1/2 at T202/Y204 whereas rapamycin decreased phosphorylation of both proteins in solid tumours suggesting that rapamycin suppresses MAPK signalling via multiple mechanisms.

Western blot was also used to investigate the effect of treatment on cellular signalling in normal tissues. Both GSK2126458 and rapamycin inhibited phosphorylation of S6 at S235/236 but did not inhibit phosphorylation of other proteins examined in the liver (Supplementary Figure 4). In the kidneys, GSK2126458 reduced phosphorylation of mTOR at S2481, Akt at S473, S6 at S235/236 and RAF1 at S259. Rapamycin also reduced phosphorylation of these proteins although to a lesser extent, except for S6 at S235/236 in the kidneys (Supplementary Figure 4).

### Effect of GSK2126458 and rapamycin on proliferation and apoptosis in renal tumours of *Tsc2*^+/-^ mice

**Figure 6:**
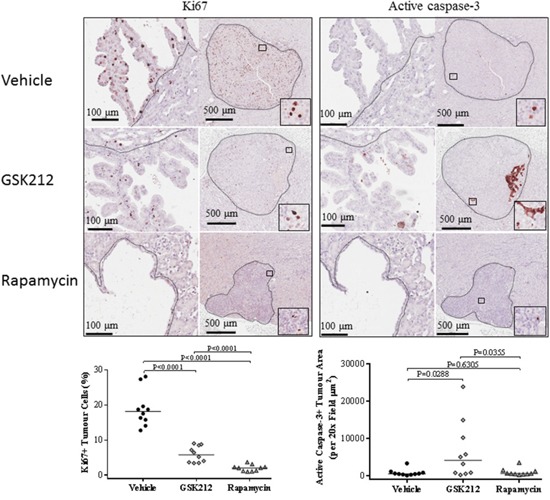
Effect of GSK2126458 and rapamycin on proliferation and apoptosis of renal tumour cells in *Tsc2*^+/-^ mice Kidney sections were prepared from 14 months old *Tsc2*^+/-^ mice after treatment and stained with antibody against Ki67 or active caspase-3 by IHC to assess proliferation or apoptosis of tumour cells. **Top left panel**: Expression of Ki67 in renal tumours. Representative sections were presented to show expression of Ki67 in tumour cells after two months treatment with vehicle, GSK2126458 and rapamycin. Black lines are scale bars. **Bottom left panel**: Percentage of Ki67-positive tumour cells. Ten fields (20x) from 10 solid tumours were selected randomly from each group and examined for quantification of Ki67-positive tumour cells using ImageJ. **Top right panel**: Expression of active caspase 3 in renal tumours. Representative sections were presented to show expression of active caspase 3 in tumour cells after two months treatment with vehicle, GSK2126458 and rapamycin. Black lines are scale bars. **Bottom right panel**: Total area of active caspase 3 positive tumour cells. Ten fields (20x) from 10 solid tumours were selected randomly from each group and examined for quantification of the total area of active caspase 3-positive tumour cells using ImageJ.

IHC-stained Ki67 was used to evaluate the effect of treatment on proliferation of renal tumour cells (Supplementary Figure 5). Ten fields (20x) from 10 solid tumours were selected randomly from each group and examined for quantification of Ki67-positive tumour cells using ImageJ. GSK2126458 and rapamycin reduced the median percentage of Ki67-positive cells from 18.13% to 5.82% and 2.07% respectively (Figure 6; Supplementary Table 3). Rapamycin inhibited proliferation of tumour cells to a greater extent than GSK2126458 (P<0.0001).

IHC-stained active caspase 3 was used to test whether treatment induced apoptosis in tumours (Supplementary Figure 6). Ten fields (20x) from 10 solid tumours were selected randomly from each group and examined for quantification of the area of active caspase 3-positive tumour cells using ImageJ. The total area of active caspase 3-positive tumour cells in each field is presented in Figure 6 and Supplementary Table 3. GSK2126458 significantly increased the median total area of active caspase 3-positive tumour cells (P=0.0288) but rapamycin did not. Similar results were observed when cleaved PARP (another apoptosis marker) was analysed by IHC (Supplementary Figure 7). To investigate mechanisms underlying increased apoptosis associated with GSK2126458 treatment, expression of p53 and phosphorylation of MDM2 at S166 were analysed by IHC. Renal lesions appeared to have a lower level of p53 and decreased phosphorylation of MDM2 in rapamycin treated mice compared to vehicle treated mice while the protein level of p53 and the phosphorylation of MDM2 appeared to be similar in lesions of GSK2126458 treated mice to those of vehicle treated mice (Supplementary Figure 8). Similar results were observed by Western analysis (Figure 5).

## DISCUSSION

In this study, we investigated the therapeutic efficacy of GSK2126458 for renal tumours in *Tsc2*^+/-^ mice in comparison to rapamycin. We demonstrated that GSK2126458 was effective for treating solid renal tumours. GSK2126458 has been used for treating xenograft tumours in immunocompromised mice and achieved significant therapeutic efficacy [8, 9]. We here used a genetically engineered *Tsc2*^+/-^ mouse model that spontaneously develops various types of renal lesions and confirmed the anti-tumour efficacy of GSK2126458 for tumours in an organ-appropriate environment. No previous comparison of anti-tumour efficacy between GSK2126458 and rapamycin has been made *in vivo*. GSK2126458 has been recently reported to be well tolerated in patients treated for multiple solid malignancy types with tumour responses and disease stabilization being seen [10]. Eight percent of these patients suffered diarrhoea and effective GSK2126458 dose remains to be determined. NVP-BEZ235, another ATP-competitive dual PI3K/mTOR inhibitor, has been used to treat renal lesions in a *Tsc2*^+/-^ mouse model on the mixed C57BL/6J:129S1/SvImJ background and appeared to be as effective as everolimus [14].

We found that GSK2126458 inhibited both mTORC1 and mTORC2 in all types of renal lesions in *Tsc2*^+/-^ mice but the inhibitory effect of GSK2126458 on mTORC1was weaker than that of rapamycin. GSK2126458 reduced phosphorylation of Akt at T308 but rapamycin did not. It remains to be determined whether this reduction in Akt phosphorylation contributes to anti-tumour efficacy. GSK216458 has been reported to activate Erk1/2 in prostate cancer cells [15]. We did not observe increased activity of Erk1/2 in GSK2126458 treated renal lesions. In contrast, we found that the phosphorylation of RAF1 at S259, a part of the Erk1/2 signalling pathway, was reduced in GSK2126458 treated solid renal tumours.

Rapamycin has been reported to induce feedback activation of Akt in various cancer cells through increased phosphorylation at S473 by mTORC2 and at T308 by PDK1 [6, 16 and 17]. We observed increased phosphorylation of Akt at S473 in over 50% of rapamycin treated cysts but not in solid tumours. No increased phosphorylation of Akt at T308 was detected in rapamycin treated solid tumours. In contrast, reduced phosphorylation of mTOR at S2481, another indicator of mTORC2 activation, was detected in rapamycin treated solid tumours. In a previous report, inhibition of mTORC1 with everolimus led to a marked increase in Erk activity in breast cancer [17]. However, we found that the Erk1/2 signalling was inhibited by rapamycin in solid renal tumours. These different observations may be partly related to the disruption of mTORC1 activation-associated feedback loops identified in renal lesions of *Tsc2*^+/-^ mice [12].

We demonstrated that both GSK2126458 and rapamycin had anti-tumour efficacy by suppressing proliferation of tumour cells but suppression by rapamycin was much stronger than by GSK2126458, probably due to its inhibition of both mTOR and Erk/1/2 activities. We found that GSK2126458 promoted apoptosis in solid renal tumours. It may be of interest to determine the possible long term benefit after cessation of GSK2126458 treatment. Previous studies have documented an increase in apoptosis and an increased ratio of p53/MDM2 in cancer cells following treatment with rapamycin [18]. We did not find that rapamycin increased apoptosis but did observe that rapamycin reduced the protein level of p53 and the phosphorylation of MDM2 in solid renal tumours. Furthermore, we did not observe a significant change in the expression of p53 and phosphorylation of MDM2 after GSK2126458 treatment, suggesting an alternative mechanism of apoptosis caused by GSK2126458. An *in vitro* study suggested that GSK2126458 increased responses of tumours to everolimus treatment [19]. It may therefore be worthwhile investigating whether combination of GSK2126458 with rapamycin could improve anti-tumour therapy through increased tumour cell death.

## MATERIALS AND METHODS

### Animal procedures

Animal procedures were performed in accordance with the UK Home Office guidelines and approved by the Ethical Review Group of Cardiff University. *Tsc2*^+/−^ BALB/c mice were described previously [11]. *Tsc2*^+/-^ litter mates were randomly allocated into 3 groups of 10, balanced for gender and of the same age. Animals were treated with vehicle, GSK2126458 (2 mg/kg, MTD) and rapamycin (4 mg/kg) for 2 months from the age of 12 months via intraperitoneal injection 5 times a week. Animals were then sacrificed for assessment of tumour burden and analysis of protein expression and phosphorylation in normal tissues and tumour samples. GSK2126458 was purchased from Medkoo, Chapel Hill, USA and rapamycin from LC Laboratories, Woburn, MA, USA. GSK2126458 of 2 mg/ml and rapamycin of 2 mg/ml were prepared respectively in 2.5% PEG-400, 2.5% Tween-80 and 2.5% DMSO.

### Histology

Assessment of tumour burden in the kidneys of mice was performed as described previously [20]. Mouse kidneys were fixed in 10% buffered formalin saline for 24 h. Fixed kidneys were processed and paraffin embedded. Six coronal sections of 5 μm were prepared at a 200 μm interval from both kidneys of each mouse. Kidney sections were haematoxylin and eosin (HE)-stained and scanned to create virtual HE slides using an Aperio system (http://www.leicabiosystems.com/?gclid=CNXN-8by4a%20UCFcINfAods3eg1w). Virtual slides were used for lesion quantification. Maximum cross-sectional whole area (i.e. including intracystic spaces) and cellular area of each renal lesion were measured, respectively, using ImageJ (http://rsbweb.nih.gov/ij). The assessment was conducted blindly with respect to treatment status. The total and cellular burdens of all lesions were estimated from total whole areas and cellular areas respectively of all lesions (cystic, papillary and solid), and the burden of solid lesions alone was estimated from the sums of their cross sectional areas.

### Immunohistochemistry (IHC) and Western blot

Immunohistochemistry (IHC) and Western blot were used to examine protein expression and phosphorylation, cell proliferation and apoptosis as described previously [12]. Kidney sections were prepared as above. SignalStain Boost Rabbit specific IHC Detection Reagent (Cell Signalling Technology, Danvers, USA) was used to stain antigens according to the kit supplier's instruction. IHC stained slides were scanned to generate virtual slides for photo capture using an Aperio system. For Western blot, extracts of normal tissues and tumour samples were prepared using AllPrep DNA/RNA/Protein Mini Kit (QIAGEN Ltd-UK, Crawley, UK). Proteins were purified according to the kit supplier's instruction. Twenty μg of protein per sample was separated on NuPAGE 4–12% Bis-Tris Gels (Fisher Scientific UK Ltd, Loughborough, UK) and transferred onto Amersham Protran Premium 0.2 or 0.45 μm nitrocellulose blotting membranes(GE Healthcare UK Ltd, Little Chalfont, UK). Blots were analysed with ECL Select Western Detection Kit (GE Healthcare UK Ltd) and signals were detected using Autochemi Imaging System (UVP, Upland, CA, USA). Horseradish peroxidise-conjugated secondary antibody against rabbit was used for Western blot (Cell Signalling Technology Danvers, USA). Primary antibodies used for IHC and Western blot were those against phosphorylated S6 ribosomal protein at S235/236, 4E-BP1 at T37/46, 4E-BP1 at S65, Akt at S473, Akt at T308, Akt substrates (RXXS*/T*), Akt substrates (RXRXXS*/T*) and Erk1/2 at T202/Y204, 4E-BP1, p53 and β-actin (Cell Signalling Technology, Danvers, MA, USA);Ki67, active caspase 3, cleaved PARP, phosphorylated PKC at T638 and RAF1 at S259 ( Abcam, Cambridge, UK); phosphrylated MDM2 at S166, mTOR at S2448 and mTOR at S2481 (Sigma-Aldrich, Dorset, UK).

### Statistical analysis

The Mann Whitney test was used to compare groups for statistical differences. P<0.05 was considered to be statistically significant. Analyses were performed using GraphPad Prism 7.01.

## SUPPLEMENTARY MATERIALS FIGURES AND TABLES



## Abbreviation

TSC: tuberous sclerosis
PI3K: phosphoinositide 3-kinase
mTOR: mechanistic target of rapamycin
mTORC1: mTOR complex 1
mTORC2: mTOR complex 2
MAPK: mitogen-activated protein kinase
AML: angiomyolipomas
IHC: immunohistochemistry
HE: haematoxylin and eosin
MDM2: Mouse double minute 2 homolog
MTD: maximum tolerated dose.
