# Supplementary material for: The dual PI3K/mTOR inhibitor GSK2126458 is effective for treating solid renal tumours in Tsc2+/- mice through suppression of cell proliferation and induction of apoptosis
Source: Oncotarget. 2017 Apr 19;8(35):58504–12. doi: 10.18632/oncotarget.17215 (PMC5601670; doi:10.18632/oncotarget.17215)
Supplement: Supplementary file 1 [file oncotarget-08-58504-s001.pdf]

The dual PI3K/mTOR inhibitor GSK2126458 is effective for treating solid renal tumours in *Tsc2*$^{+/-}$ mice through suppression of cell proliferation and induction of apoptosis

SUPPLEMENTARY FIGURES AND TABLES

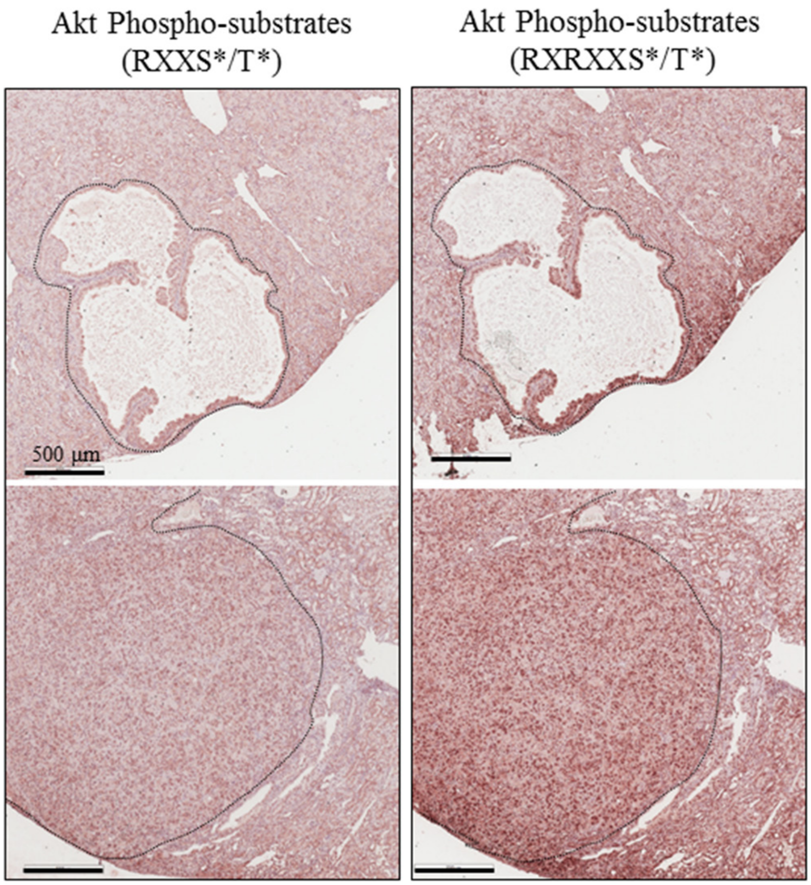

Supplementary Figure 1: Phosphorylation of Akt substrates in renal tumours of *Tsc2*$^{+/-}$ mice. Kidney sections were prepared from 14 months old *Tsc2*$^{+/-}$ mice and stained by IHC. Representative kidney sections were presented to show increased phosphorylation of Akt substrates in renal tumours. Scale bars are 500 µm.

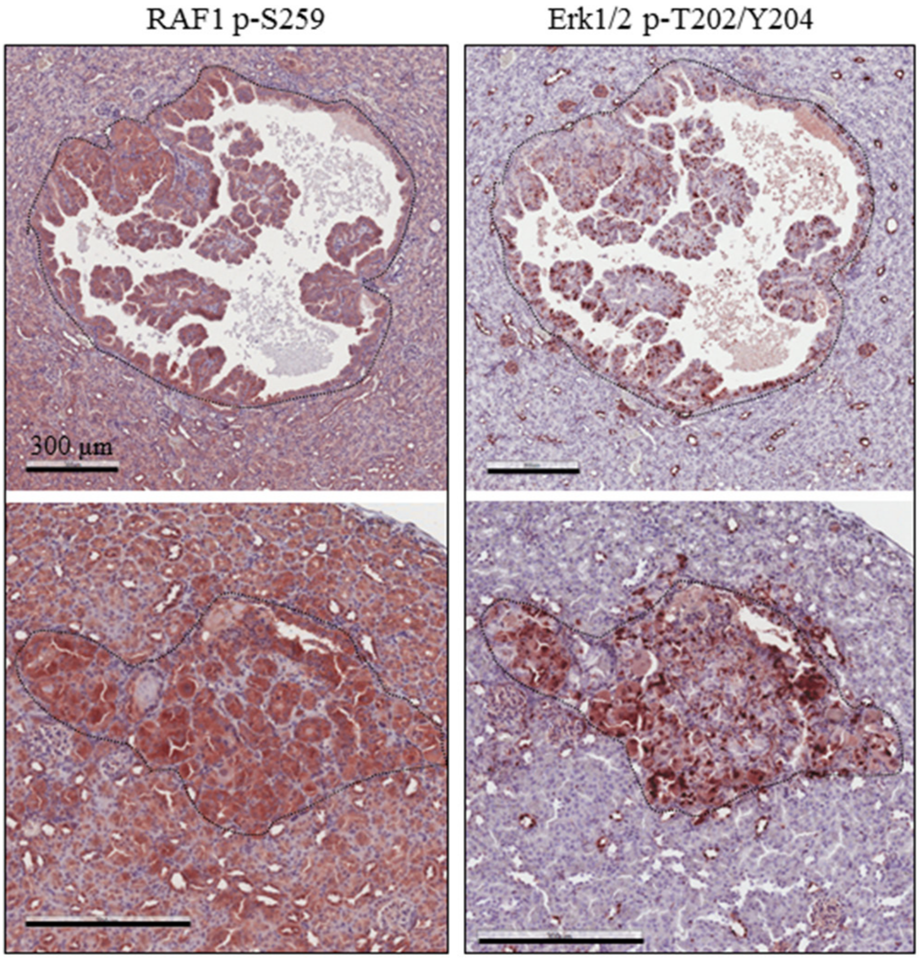

Supplementary Figure 2: MAPK signalling in renal tumours of *Tsc2*$^{+/-}$ mice. Kidney sections were prepared from 14 months old *Tsc2*$^{+/-}$ mice and stained by IHC. Representative kidney sections were presented to show increased phosphorylation of RAF1 at S259 and Erk1/2 at T202/Y204 in renal tumours. Scale bars are 300 µm.

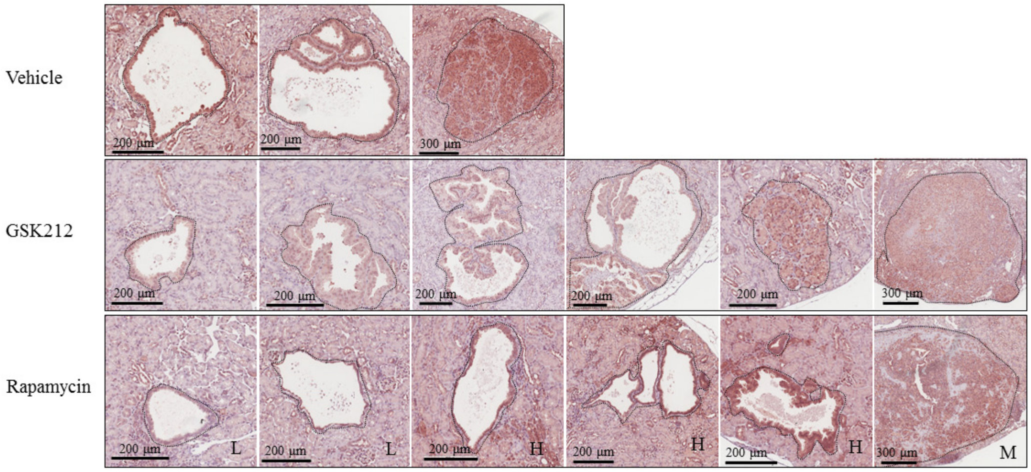

Supplementary Figure 3: Effect of GSK2126458 and rapamycin on phosphorylation of Akt at S473 in renal tumours of *Tsc2*$^{+/-}$ mice. Kidney sections were prepared from 14 months old *Tsc2*$^{+/-}$ mice treated by vehicle, GSK2126458 or rapamycin and stained by IHC. Representative kidney sections were presented to show reduced phosphorylation of Akt at S473 in GSK2126458 treated renal tumours but varied phosphorylation of Akt at S473 in rapamycin treated tumours. Around 57% (20/35) rapamycin treated cysts exhibited highly phosphorylated Akt at S473 as indicated by "H". L, low phosphorylation of Akt at S473; M, medium phosphorylation of Akt at S473; H, high phosphorylation of Akt at S473. Black lines are scale bars.

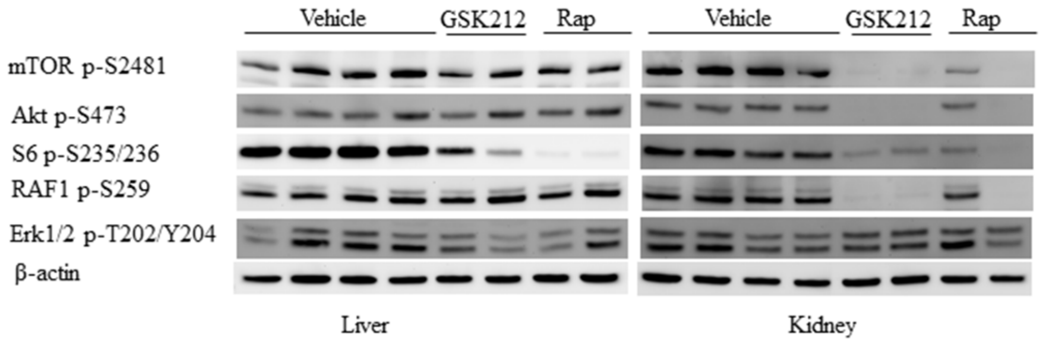

Supplementary Figure 4: Effect of GSK2126458 and rapamycin on mTOR/MAPK signalling in normal tissues of *Tsc2*$^{+/-}$ mice. Western blot was used to analyse mTOR/MAKP signalling. Proteins were prepared from normal tissues of *Tsc2*$^{+/-}$ mice treated for one month with vehicle, GSK2126458 or rapamycin. Beta-actin was used as a loading control. Representative Western blots were presented to show phosphorylation of mTOR at S2481, Akt at S473, S6 at S235/236, RAF1 at S256 and Erk1/2 at T202/Y204.

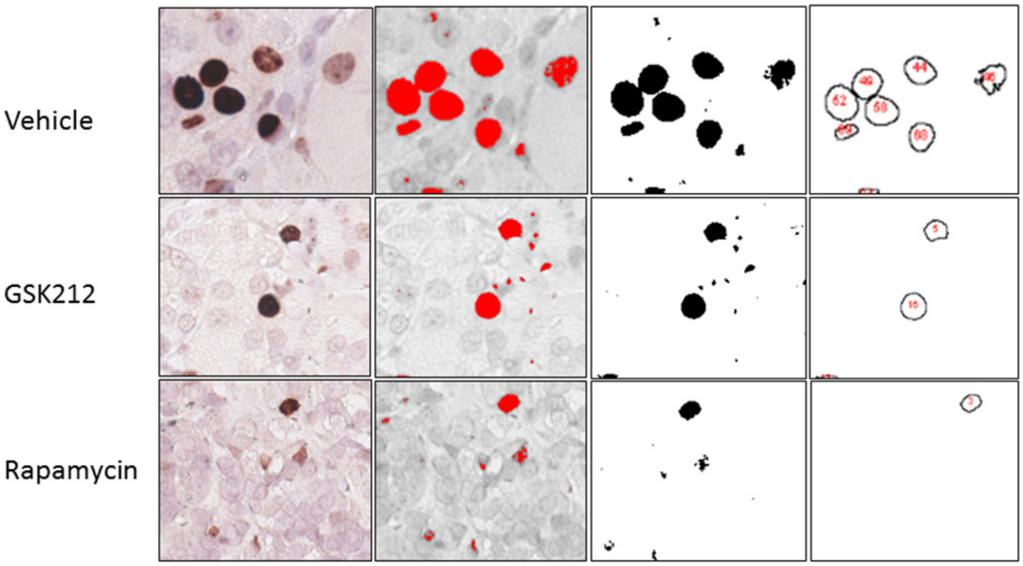

Supplementary Figure 5: Quantification of Ki67 expression in renal tumours of *Tsc2*$^{+/-}$ mice. Kidney sections were prepared from 14 months old *Tsc2*$^{+/-}$ mice treated by vehicle, GSK2126458 or rapamycin and stained by antibody against Ki67 with IHC. Images of IHC stained sections were processed and Ki67-positive tumour cells were counted using ImageJ.

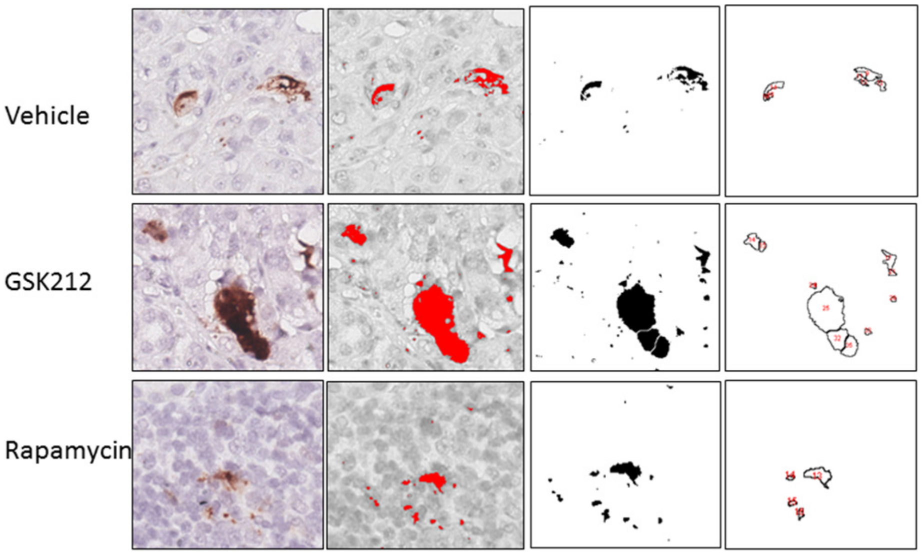

Supplementary Figure 6: Quantification of active caspase 3 expression in renal tumours of *Tsc2*$^{+/-}$ mice. Kidney sections were prepared from 14 months old *Tsc2*$^{+/-}$ mice treated by vehicle, GSK2126458 or rapamycin and stained by antibody against active caspase 3 with IHC. Images of IHC stained sections were processed and total area of active caspase 3-positive tumour cells were measured using ImageJ.

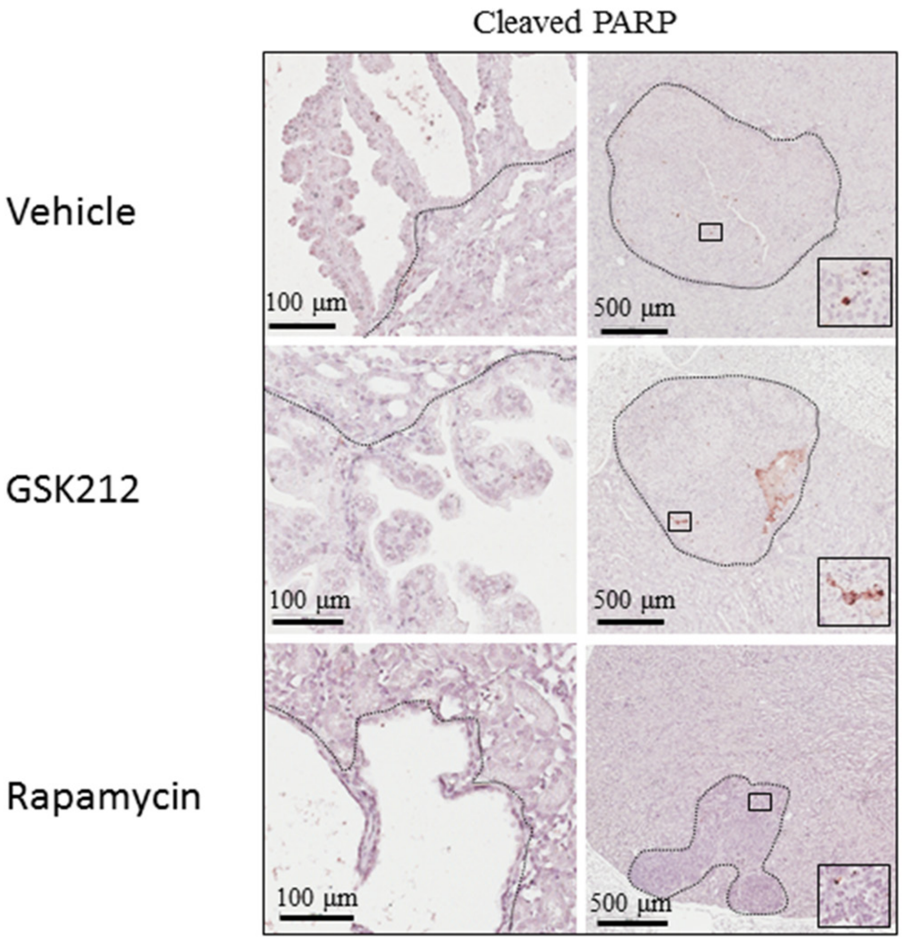

Supplementary Figure 7: Expression of cleaved PARP in renal tumours of *Tsc2*$^{+/-}$ mice. Kidney sections were prepared from 14 months old *Tsc2*$^{+/-}$ mice treated by vehicle, GSK2126458 or rapamycin and stained by antibody against cleaved PARP with IHC. Black lines are scale bars.

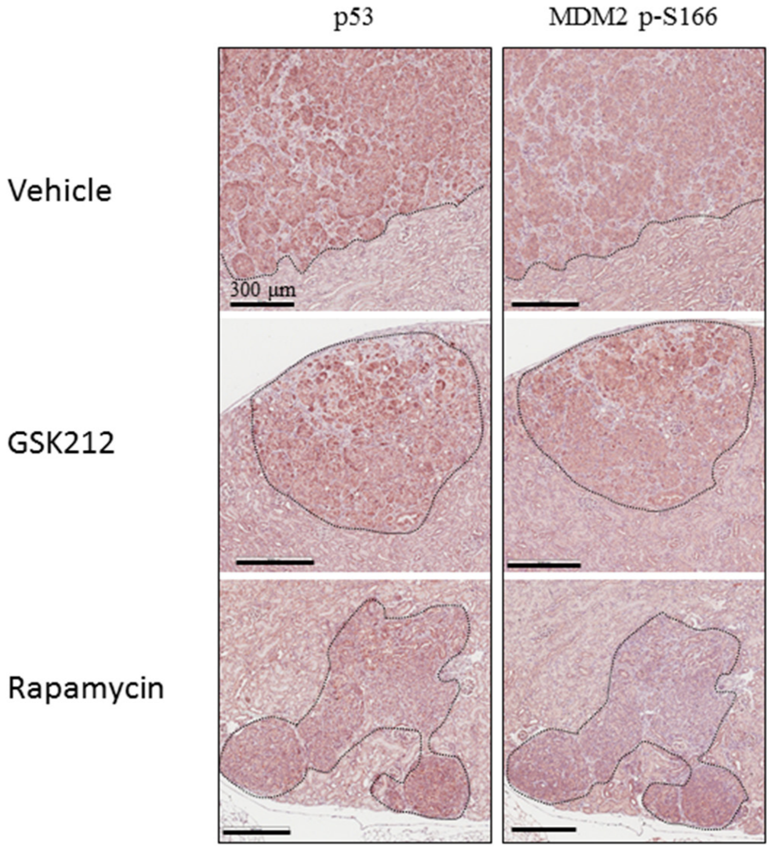

Supplementary Figure 8: Expression of p53 and phosphorylation of MDM2 at S166 in renal tumours of *Tsc2*$^{+/-}$ mice. Kidney sections were prepared from 14 months old *Tsc2*$^{+/-}$ mice treated by vehicle, GSK2126458 or rapamycin and stained by IHC. Representative kidney sections were presented to show expression of p53 and phosphorylation of MDM2 at S166 in renal tumours. Scale bars are 300 μm.

Supplementary Table 1: Comparison of solid tumours in Tsc2+/- mice by histological analysis
(Mann Whitney test)

A. Lesion number (solid tumours)

Treatment*	Number of mice	Median	Range	P (compared with Vehicle)	P (compared with rapamycin)
Vehicle	10	4.0	1-7		
GSK2126458	10	2.0	0-7	0.0404	0.1206
Rapamycin	10	1.0	0-3	0.0003	

B. Lesion size (solid tumours)

Treatment*	Number of mice	Median (mm^2)	Range (mm^2)	P (compared with Vehicle)	P (compared with rapamycin)
Vehicle	10	3.34	0.19-36.28		
GSK2126458	10	0.33	0-11.91	0.0281	0.0507
Rapamycin	10	0.02	0-1.22	<0.0001	

C. Lesion cellular area (solid tumours)

Treatment*	Number of mice	Median (mm^2)	Range (mm^2)	P (compared with Vehicle)	P (compared with rapamycin)
Vehicle	10	3.25	0.18-35.3		
GSK2126458	10	0.32	0-11.73	0.0345	0.0507
Rapamycin	10	0.02	0-1.22	<0.0001	

* Treatment was started from 12 month old and continued for two months.

Supplementary Table 2: Comparison of all lesion types in $Tsc2^{+/-}$ mice by histological analysis
(Mann Whitney test)

A. Lesion number (all lesions-cystic, papillary and solid)

Treatment*	Number of mice	Median	Range	P (compared with Vehicle)	P (compared with rapamycin)
Vehicle	10	36.0	25-43		
GSK2126458	10	28.5	21-39	0.0448	0.0002
Rapamycin	10	14.5	3-30	<0.0001	

B. Lesion size ((all lesions-cystic, papillary and solid)

Treatment*	Number of mice	Median (mm^2)	Range (mm^2)	P (compared with Vehicle)	P (compared with rapamycin)
Vehicle	10	9.12	6.51-44.66		
GSK2126458	10	6.27	2.13-19.57	0.0185	0.0039
Rapamycin	10	1.82	0.07-6.46	<0.0001	

C. Lesion cellular area ((all lesions-cystic, papillary and solid)

Treatment*	Number of mice	Median (mm^2)	Range (mm^2)	P (compared with Vehicle)	P (compared with rapamycin)
Vehicle	10	5.11	2.49-38.65		
GSK2126458	10	2.64	0.88-15.33	0.0433	0.0015
Rapamycin	10	0.35	0.02-3.49	<0.0001	

* Treatment was started from 12 month old and continued for two months.

Supplementary Table 3: Effect of treatment on proliferation and apoptosis of renal tumours in Tsc2$^{+/-}$ mice (Mann Whitney test)

A. Percentage of tumour cells positive for Ki67

Treatment*	Number of fields examined (20x)**	Median (%)	Range (%)	P (compared with Vehicle)	P (compared with rapamycin)
Vehicle	10	18.130	12.74-28.08		
GSK2126458	10	5.820	3.43-9.04	<0.0001	<0.0001
Rapamycin	10	2.070	0.99-3.68	<0.0001	

B. Area of tumour cells positive for active caspase 3

Treatment*	Number of fields examined (20x)**	Median (μm^2)	Range (μm^2)	P (compared with Vehicle)	P (compared with rapamycin)
Vehicle	10	549.548	96.117-3327.086		
GSK2126458	10	4121.250	245.266-23861.254	0.0288	0.0355
Rapamycin	10	587.254	230.233-3508.020	0.6305	

* Treatment was started from 12 month old and continued for two months.

** Ten high-powered fields (20x) from 10 solid tmours (1 field each) were analysed per group.
